# Analysis of Factors Influencing Outcomes in Preterm Infants With Necrotizing Enterocolitis

**DOI:** 10.3389/fped.2022.768107

**Published:** 2022-05-13

**Authors:** JinBao Han, Gang Liu, MengNan Yu, Guang Li, JianYing Cao, Lian Duan, LiuMing Huang

**Affiliations:** ^1^Department of Neonatal Surgery, Senior Department of Pediatrics, the Seventh Medical Center of PLA General Hospital, Beijing, China; ^2^National Engineering Laboratory for Birth Defects Prevention and Control of Key Technology, Beijing, China; ^3^Beijing Key Laboratory of Pediatric Organ Failure, Beijing, China

**Keywords:** necrotizing enterocolitis, non-perforation and perforation, surgery, Bell stage, mortality

## Abstract

**Background:**

To explore the surgical outcomes between patients with perforated and non-perforated neonatal necrotizing enterocolitis (NEC) and identify indications for surgical intervention.

**Methods:**

The surgical outcomes of 271 children with NEC admitted to the Seventh Medical Center of Chinese PLA General Hospital between August 2009 and August 2020 were retrospectively analyzed. The patients were divided into the non-perforated and perforated groups. The preoperative factors, including gestational age, birth weight, intrauterine infection, cholestasis, platelet change, white blood cell count, and C-reactive protein level were compared between the two groups, along with postoperative factors including infection status, complications, enteral and parenteral nutrition time, ICU time, ventilator use time, and intestinal necrosis length. Bell staging was performed for the two groups and the mortality of different Bell stages was explored. The risk of death and predisposing factors of patients with NEC were analyzed.

**Results:**

In total, 271 children undergoing surgery were included in this study. A total of 188 children were observed without perforation, including 57 deaths (30.3%), and 83 children with perforation, including 24 deaths (28.9%). Preoperative cholestasis and time from NEC diagnosis to surgery were significantly different between the two groups (*P* < 0.05). Postoperative factors, including parenteral nutrition time (32 [3–94] days vs. 23 [1–53] days), enteral nutrition time (27 [0–86] days vs. 18 [0–81] days), NICU time (44 [5–125] days vs. 29 [1–92] days), and length of intestinal necrosis (15 [0–92] cm vs. 10 [2–70] cm), were significant. The mortality rate of patients with Bell stage IIIA was higher than that of patients with Bell stage IIIB. A total of 81 patients had 30-day postoperative mortality (57 non-perforated cases). Multivariate Cox regression analysis showed that non-perforation was a poor prognostic factor for survival outcome (hazard ratio 2.288, 95% confidence interval [1.329-3.940], *P* = 0.003).

**Conclusions:**

Preterm infants in the non-perforated group had more serious complications and had a longer recovery time after surgery. Bell staging is not accurate in diagnosing severe NEC that needs surgical intervention.

## Introduction

Necrotizing enterocolitis (NEC) is the most common surgical emergency in neonates and is classically a disease of prematurity (1), with approximately 90% of cases diagnosed in preterm infants (2). Approximately 40% of neonates with NEC require surgery, which are at a high risk of death or require long-term hospitalization (3). With the improvement in the level of care in the intensive care unit, the survival rate of NEC is gradually increasing. Due to the rapid progress of NEC, the mortality rate of patients with NEC is higher when surgery is required. To date, the only absolute indication for surgical intervention in infants with NEC is bowel perforation. However, the surgical indications for non-perforated NEC remain controversial, and many clinicians consider clinical deterioration in patients receiving maximal medical therapy to be an indication for surgery for non-perforated NEC. The presence of pneumoperitoneum on radiographic images indicates perforation and is the only absolute criterion for surgery (4). In the classic Bell staging, perforation is classified as stage IIIB, which is considered the most serious stage of the disease. However, it seems that the outcome of patients with perforation is not worse than that of patients without perforation. This study aimed to analyze the outcomes of preterm infant surgery under two different surgical conditions, non-perforation and perforation, to provide evidence for exploring the timing of surgical NEC intervention in preterm infants.

## Materials and Methods

This study (retrospective data analysis) was approved by the Ethics Committee of the Seventh Medical Center of PLA General Hospital. Patient confidentiality was protected by deidentifying the data prior to analysis. A total of 271 children with surgical NEC were admitted in our department between August 2009 and August 2020. Patients with spontaneous intestinal perforation (isolated intestinal perforation without histological or clinical evidence of necrotizing enterocolitis), secondary intestinal necrosis (Necrosis caused by other malformation), or perforation caused by other diseases were excluded. Those without perforation were excluded from cases with peritoneal drain as initial surgery or local repair, and only those with necrotic bowel in need of resection or clip and drop-back were selected. NEC staging refers to the revised modified Bell staging standard for neonatal NEC (5). Children with congenital gastrointestinal malformations and genetic metabolic diseases were excluded from both groups.

Before the operation, the children whose abdominal radiographs showed pneumoperitoneum were classified into perforation group, and the rest in the non-perforated group. Regarding observation indicators, in the perforation group, surgical treatment was generally completed within 6 hours after the perforation was cleared. The preoperative indications for surgery in the non-perforated group were based on the Duke abdominal X-ray score (DAAS) (consider surgical treatment when scores >7) (6), seven metabolic disorders (MD7) (≥4 indicators,consider surgical) (7, 8), and white blood cell/platelet level changes (We used the formula:ln [P/(1 – P)] = 2.801 – 0.207WBC2 – 0.008PLT2. We set *P* = 0.55, and consider surgical treatment when P > 0.55) (9), specifically, clinical manifestations that have not improved even with medical management, persistent abdominal distension, obvious symptoms of peritonitis, abdominal wall varicose veins, redness of the abdominal wall skin, and a palpable abdominal mass combined with changes in patients' laboratory infection indicators (levels of leukocytes/platelets, CRP, total blood bilirubin, etc.) and radiographic examination of the vertical abdominal plain film showing obvious signs of gas accumulation between the intestinal walls, gas accumulation in the portal vein, and fixed bowel loops. Before 2017, the assessment systems we used for the severity of the disease were DAAS and MD7, after 2017, the evaluation system for the severity of the disease is DAAS, MD7, & white blood cells, platelets count. The patients match any of these indicators, which to consider surgical treatment. The indications were consistent in all patients, and immediate surgical treatment was provided. Preoperative statistical data included gestational age; birth weight; heart malformation; prenatal intrauterine infection; laboratory indicators (blood biochemistry, routine blood test, CRP test, etc.); surgical methods for the non-perforated group including intestinal resection and enterostomy (143 cases), clip and drop-back (21 cases), and intestinal resection and anastomosis (24 cases); and the operative method for the perforation group including bowel resection and enterostomy (83 cases). The length of necrotic intestines in the two groups was recorded. Postoperative statistics included mortality (30-day postoperative mortality), severe postoperative complications (acute respiratory distress syndrome, intracranial hemorrhage, acute renal insufficiency), postoperative infection time, parenteral nutrition time, enteral nutrition time, ICU stay, and time of ventilator use. Postoperative time refers to the time from the surgery to discharge from the hospital. Through the establishment of Cox regression model, including gestational age, body weight, postoperative infection time, postoperative enteral nutrition time and parenteral nutrition time, length of NICU time, which association with postoperative survival. The risk of death was compared between the non-perforated group and the perforated group.

### Statistical Analyses

Statistical analyses were performed using SPSS Statistics for Windows (version 19.0; IBM Corporation, Armonk, NY, USA). Measurement data that observed a normal distribution were represented by mean ± SD, and the independent sample t-test was used for comparison between the two groups. Non-normally distributed measurement data were represented by median (minimum, maximum), and the Wilcoxon ranked-sum test of independent samples was used for comparison between groups. The chi-square test was used to compare the count data between groups. Survival was estimated using the Kaplan–Meier method and statistically compared using the log-rank test. Hazard ratios (HRs) were calculated, and multivariate analysis was performed using Cox's proportional hazards model. Statistical significance was set at *P* < 0.05.

## Results

### Pre-Operative Characteristics in Perforated and non-Perforated Cases

A total of 271 preterm infants underwent NEC surgery: males, 171 cases; females, 100 cases, of which 188 cases were in the non-perforated group before the operation. According to the modified Bell criteria for the diagnosis of NEC (5), 16 cases were in stage IIA, 99 cases in stage IIB, and 73 cases in stage IIIA. All 83 patients in the perforation group were diagnosed with stage IIIB disease.

The results showed that gestational age, birth weight, intrauterine infection, patent ductus arteriosus, white blood cell count, thrombocytopenia, and CRP level were not statistically different between the two groups (*P* > 0.05). Preoperative cholestasis was observed in 143 cases (76.1%) in the non-perforated group and 48 cases (57.8%) in the perforated group, and the difference was statistically significant (*P* = 0.002). The time from NEC diagnose to surgery were 29 (4–82) hin the non-perforated group and 22 (3–76) h in the perforated group, and the difference was statistically significant (*P* = 0.023) (Table 1).

**Table 1:** Comparison of preoperative factors in the non-perforated and the perforated groups [n (%), M (min, max)].

	**Total *n* = 271**	**The non-perforated group *n* = 188**	**The perforated group *n* = 83**	***x^**2**^*/*Z***	***P*-value**
Gestational age, week	31 (25–36)	30 (25–36)	31 (26–36)	−1.477	0.140
Birth weight, g	1650 (500–3485)	1600 (610–3485)	1860 (500–3160)	−0.805	0.421
Cholestasis^a^	191 (70.5)	143 (76.1)	48 (57.8)	9.2	0.002*
time from NEC diagnosis to surgery, h	26 (3–82)	29 (4–82)	22 (3–76)	−2.267	0.023*
PDA^b^	103 (38)	68 (36.2)	35 (42.2)	0.879	0.348
Intrauterine infection	186 (68.6)	125 (66)	61 (73)	1.31	0.252
Thrombocytopenia^c^	130 (21–404)	139 (21–404)	128 (28–379)	−0.051	0.959
Leukocyte^d^	6.6 (1.9–29.4)	6.6 (1.9–29.4)	6.1 (2.3–26.4)	−1.471	0.141
CRP ^e^	59 (1–292)	60 (1–234)	59 (1–292)	−0.872	0.383

a *Cholestasis: total bilirubin is elevated, direct bilirubin > 17μmol/L;*

b *PDA: Patent Ductus Arteriosus;*

c *Platelets decrease below normal value: platelet count < 1001 × 0^9^/L;*

d White Blood Cell normal value 4.0–10.0 * 104^9^/L;

e *, CRP normal value 0–8mg/L;*

* *There is a statistical difference*.

### Post-Operative Results of non-Perforated and Perforated Cases

The death rate (30 days postoperative mortality) was higher in the non-perforated group (57/188 deaths [30.3%]) than that in the perforated group (24/83 deaths [28.9%]); however, the difference was not statistically significant. No statistically significant difference in severe postoperative complications (68 cases [36.4%] in the non-perforated group and 29 cases [34.9%] in the perforated group) and ventilator use time (4 [1–24] days vs. 3 [1–28] days) was observed (*P* > 0.05). The differences in parenteral nutrition time (32 [3–94] days vs. 23 [1-53] days), length of intestinal necrosis (15 [0–92] cm vs. 10 [2–70] cm), enteral nutrition time (27 [0–86] days vs. 18 [0–81] days), and NICU stay (44 [5–125] days vs. 29 [1–92] days) were statistically significant between the two groups (*P* < 0.05). The postoperative recovery time in the non-perforated group was significantly longer than that in the perforated group (Table 2).

**Table 2:** Post-operative results between two groups [n (%), M (min, max)].

	**Total *n* = 271**	**The non-perforated group *n* = 188**	**The perforated group *n* = 83**	***x^**2**^*/*Z***	***P*-value**
Death (30-day mortality), n	81 (29.8)	57 (30.3)	24 (28.9)	0.003	0.956
Serious postoperative complications, *n* ^e^	97 (35.8)	68 (36.4)	29 (34.9)	0.42	0.517
Postoperative infection time, d ^f^	13 (1–49)	15 (1–49)	12 (1–27)	−1.238	0.216
Parenteral nutrition time, d ^g^	29 (1–94)	32 (3–94)	23 (1–53)	−2.923	0.003*
Enteral nutrition time, d ^h^	25 (0–86)	27 (0–86)	18 (0–81)	−2.159	0.031*
Length of NICU time, d	40 (1–125)	44 (5–125)	29 (1–92)	−2.710	0.007*
Ventilator use time, d	4 (1–28)	4 (1–24)	3 (1–28)	−1.854	0.064
Length of intestinal necrosis, cm	12 (0–92)	15 (0–92)	10 (2–70)	−2.464	0.014*

e *Severe complications: at least one of acute respiratory distress syndrome, intracranial hemorrhage, and acute renal insufficiency;*

f *Duration of postoperative infection: from the beginning of use to the degradation of antibiotics when the infection improves;*

g *Parenteral nutrition time: from the start of intravenous nutrition to the stop;*

h *Enteral nutrition time: from start to full dose;*

* *There is a statistical difference*.

### Bell Staging and Mortality

In the Bell staging, all preterm infants with perforation were classified as stage IIIB, and the rest were identified as non-perforated. The mortality rate was 18.8% (3/16) in stage IIA, 21.2% (21/99) in stage IIB, 45.2% (33/73) in stage IIIA, and 28.9% (24/83) in stage IIIB patients. The mortality rate of patients with stage IIIA was higher (Table 3).

**Table 3:** Death in different Bell stages [n (%)].

**Bell stage**	**IIA**	**IIB**	**IIIA**	**IIIB**
Cure	13	78	40	59
Death	3 (18.8)	21 (21.2)	33 (45.2)	24 (28.9)
*X^2^*	0.125	0.437	4.445	-
*P-value*	0.612	0.513	0.035	-

### Multivariate Analysis of Survival

**Figure 1:**
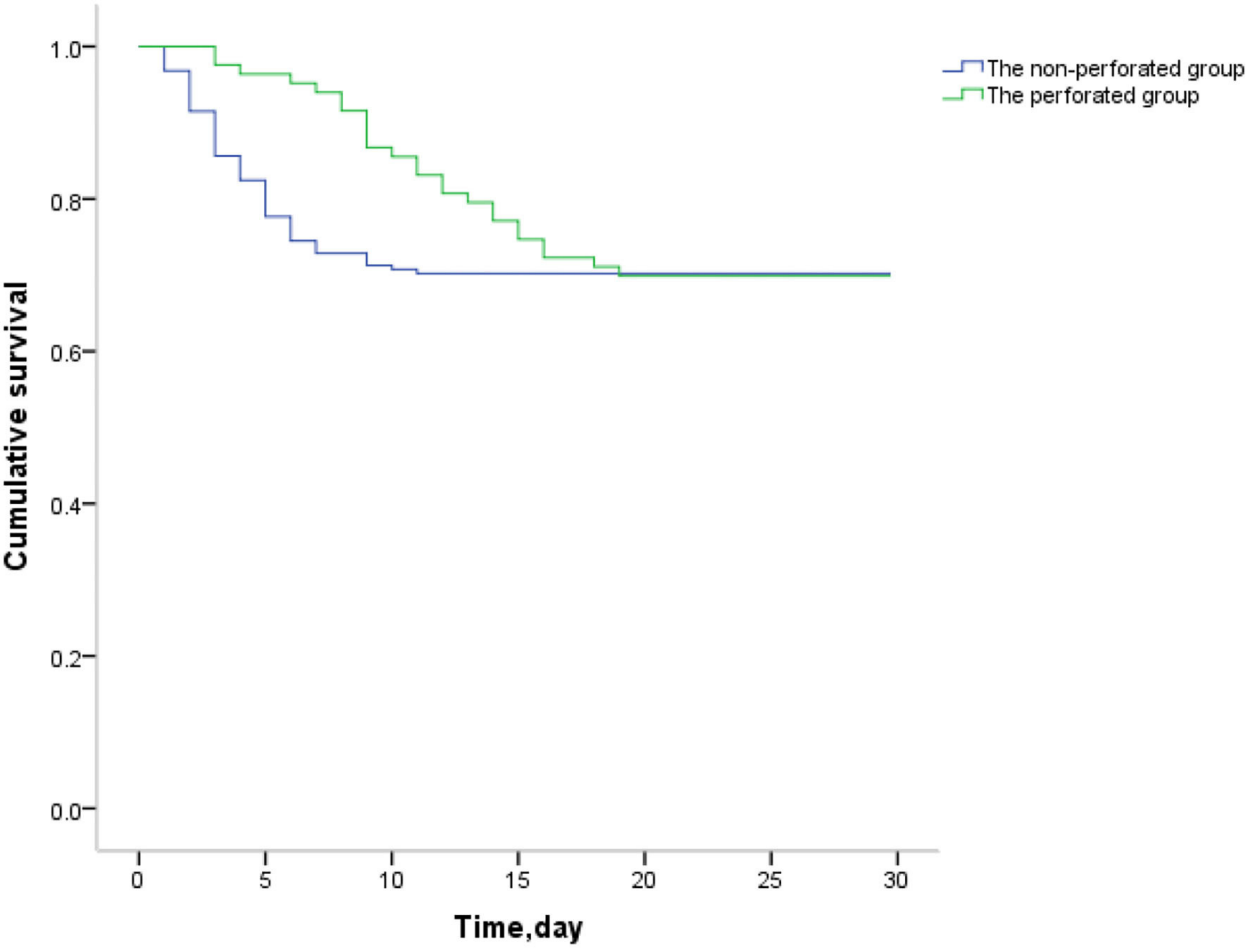
Survival at 30 days outcome of the non-perforated vs. the perforated groups. The mortality occurred earlier in the non-perforated group than the perforated group.

It was found that the risk of death in the non-perforated group was twice that of the perforated group. (HR, 2.288; 95% confidence interval [CI], 1.329–3.940; *P* = 0.003) (Table 4). From the COX regression graph, it is found that the slope of the non-perforated group is twice that of the perforated group (Figure 1).

**Table 4:** Multivariate proportional hazards analysis for survival at 30 days.

**Variables**	**Hazard ratio (95%, CI)**	***P*-value**
Gestational age, wk	0.918 (0.819–1.030)	0.145
Birth weight, g	1.000 (0.999–1.001)	0.613
Length of intestinal necrosis, cm	1.017 (1.010–1.024)	0.000*
Postoperative infection time, d	0.937 (0.897–0.979)	0.003*
Parenteral nutrition time, d	1.031 (0.981–1.083)	0.225
Enteral nutrition time, d	0.916 (0.882–0.952)	0.000*
Length of NICU time, d	0.986 (0.948–1.025)	0.478
The non–perforated group		
(VS. the perforated group)	2.288 (1.329–3.940)	0.003*

* *P <0.05; CI, confidence interval*.

## Discussion

Despite the continuous improvement in the level of treatment, NEC remains a serious life-threatening gastrointestinal disease in premature infants and is accompanied by serious complications (10, 11). Retrospective studies have shown that approximately 20–60% of preterm infants with NEC require surgery (12–14). Despite improvements in neonatal care, overall mortality (32%) for advanced NEC has not changed over the past 10 years (15). Due to the rapid progress of NEC, the mortality rate is always ranked first in the neonatal care unit. Multicenter studies have shown that there were 8,935 extremely low birth weight cases of NEC surgery in the United States, with a mortality rate of 35% (16, 17). At present, there is no consensus regarding surgical indication in children without perforation. In this group of studies, we found that the severity of the disease in the non-perforated group was significantly more severe than that of the perforated group. Strategies for establishing and applying these criteria would include the development of highly sensitive specific biomarkers and new techniques for detecting factors that confer a predisposition to NEC (18).

Preterm infants in the non-perforated group were more severely ill than those in the perforated group. The length of the necrotic intestine during surgery may be an important factor. In addition, gangrene occurs in the ischemic intestine, and more toxins and bilirubin may be absorbed into the blood, which may lead to a systemic inflammatory response and cholestasis. So that it takes longer time for patients without perforation to recover. Patients without perforation often have unclear indications for surgery. Timing of surgery varies in such cases depending on the surgeon's judgment with potential for delay in prompt optimal management. Gfroerer et al. (19) found that in the surgical treatment of 57 cases of NEC in preterm infants with extremely low birth weight, early laparotomy is safe and effective. Can the advancement of operation time effectively reduce the mortality rate? There is still a lack of international consensus or guidelines for the establishment of indications for non-perforated NEC surgery that guide clinical practice. The methods currently used to guide the indications for non-perforated NEC surgery also include various biomarkers, such as intestinal fatty acid-binding protein (20, 21) and serum amyloid A (22, 23). However, actual clinical practice also includes many factors such as gestational age, weight, degree of infection, abnormal bacterial colonization, excessive intestinal feeding, release of inflammatory mediators, and heart malformations affecting the intestinal circulation. But the aforementioned methods for judging the indications seem insufficient.

Bell staging is currently a widely accepted staging method that reflects NEC severity. According to Bell staging, preterm infants with perforation are classified as stage IIIB, and it is generally believed that patients with perforation have more severe disease. However, from a clinical observation, patients with NEC who require surgical treatment without perforation tend to progress faster and develop septic shock earlier. As far as surgical is concerned, the Bell staging does not reflect the severity of the disease very well, a more reliable stage is needed to judge the course of the disease (18). If the patients miss the appropriate opportunity for surgery may be lead to a serious poor prognosis. The mortality rates of the two groups were found to be similar. From the number of deaths for each stage, it can be seen that the mortality rate of patients with stage IIIA is the highest, where the condition is relatively more serious. It may be because of the remaining intestine is too short, which leads to difficulty in feeding that caused a long postoperative recovery time. The non-perforated group took significantly longer time to recover than that in the perforated group. In addition, serious complications were more likely to occur after surgery, suggesting that the severity of the disease in the non-perforated group was more serious than that in the perforated group.

In the comparative analysis of survival function, it was found that the factors affecting death were associated with the length of the necrotic bowel, postoperative infection time, and the time of enteral nutrition, which were significantly different. Regarding risk factor analysis, from the Cox regression model of the 30-day postoperative mortality in the non-perforated group was identified as a poor prognostic factor. This also explains why the condition of NEC patients after surgery in the non-perforated group was more serious than that in the perforated group.

After exclusion of patients with suspected NEC (Bell stage I) in our study, we only examined those with advanced NEC that required surgery. The retrospective nature and single-center design with a relatively small sample size are limitations of this study. Further multicenter prospective studies are needed to validate the effectiveness and generalizability of these surgical approaches.

## Conclusion

Infants with surgical NEC in the non-perforated group were more prone to bowel necrosis, and their mortality rate was higher than that in the perforated group. The non-perforated group during the postoperative recovery process had more serious complications than that in the perforated group. Bell staging is not accurate in diagnosing severe NEC that needs surgical intervention.

## Data Availability Statement

The original contributions presented in the study are included in the article/supplementary material, further inquiries can be directed to the corresponding author/s.

## Ethics Statement

The studies involving human participants were reviewed and approved by this study was approved by the Ethics Committee of the Seventh Medical Center of PLA General Hospital and, as a retrospective study, data have been managed keeping personal information concealed. Written informed consent from the participants' legal guardian/next of kin was not required to participate in this study in accordance with the national legislation and the institutional requirements.

## Author Contributions

JH, and LH contributed to conception and design of the study. JH, GL, MY, GL, JC, and LD performed the data analysis. JH, GL, and LH interpreted results, wrote, and finalized the manuscript. JH GL, and LH reviewed and revised the manuscript. All authors contributed to the article and approved the submitted version.

## Funding

This project has been funded by the Health Bureau of the Logistics Support Department of the Military Commission (Grant no. 21JSZ18).

## Conflict of Interest

The authors declare that the research was conducted in the absence of any commercial or financial relationships that could be construed as a potential conflict of interest.

## Publisher's Note

All claims expressed in this article are solely those of the authors and do not necessarily represent those of their affiliated organizations, or those of the publisher, the editors and the reviewers. Any product that may be evaluated in this article, or claim that may be made by its manufacturer, is not guaranteed or endorsed by the publisher.

## Abbreviations

CI: Confidence Interval
DAAS: Duke abdominal X-ray score
HR: Hazard ratio
MD7: Seven Metabolic Disorders
NEC: Necrotizing Enterocolitis
NICU: Neonatal Intensive Care Unit.

## References

[1] Lin PW Stoll BJ. Necrotising enterocolitis. Lancet. (2006) 368:1271–83. 10.1016/S0140-6736(06)69525-1 17027734

[2] Ostlie DJ Spilde TL St Peter SD Sexton N Miller KA Sharp RJ. Necrotizing enterocolitis in full-term infants. J Pediatr Surg. (2003) 38:1039–42. 10.1016/S0022-3468(03)00187-8 12861534

[3] Yanowitz TD Sullivan KM Piazza AJ Brozanski B Zaniletti I Sharma J. Does the initial surgery for necrotizing enterocolitis matter? Comparative outcomes for laparotomy vs. peritoneal drain as initial surgery for necrotizing enterocolitis in infants <1000 g birth weight. J Pediatr Surg. (2019) 54:721–17. 10.1016/j.jpedsurg.2018.12.010 30765157

[4] Hong CR Han SM Tom J. Surgical considerations for neonates with necrotizing enterocolitis. Sem Fetal Neonat Med. (2018) 23:420–5. 10.1016/j.siny.2018.08.007 30196017

[5] Bell MJ Ternberg JL Feigin RD Keating JP Marshall R Barton L. Neonatal necrotizing enterocolitis. Therapeutic decisions based upon clinical staging. Ann Surg. (1978) 187:1–7. 10.1097/00000658-197801000-00001 413500 PMC1396409

[6] Coursey CA Hollingsworth CL Wriston C Beam C Rice H Bisset G. Radiographic Predictors of Disease Severity in Neonates and Infants With Necrotizing Enterocolitis. Am J Roentgenol. (2009) 193:1408–13. 10.2214/AJR.08.2306 19843760

[7] Tepas JJ 3rd Sharrna R Leaphart CL Celso BG Pieper P Esquivia-Lee V. Timing of surgical intervention in necrotizing entrocolitis can be determined by trajectory of metabolic derangement. J Pediatr Surg. (2010) 45:310–4. 10.1016/j.jpedsurg.2009.10.069 20152342

[8] Tepas JJ 3rd Leaphart CL Phumley D Sharma R Celso BG Pieper P et a1. Trajectory of metabolic derangement in Infants with Necrotizing Entrocolitis Should Drive Timing and Technique of Surgical Intervention. J Am Coll Surg. (2010) 210:847–52. 10.1016/j.jamcollsurg.2010.01.008 20421063

[9] Mengnan Y Gang L Zhichun F Huang L. Combination of plasma white blood cell count, platelet count and C-reactive protein level for identifying surgical necrotizing enterocolitis in preterm infants without pneumoperitoneum. Pediatr Surg Int. (2018) 34,945–50. 10.1007/s00383-018-4305-6 30027466

[10] Knell J Han SM Jaksic T Modi BP. Current status of necrotizing enterocolitis. Curr Probl Surg. (2019) 56:11–38. 10.1067/j.cpsurg.2018.11.005 30691547

[11] Murphy T Yang S Tucker R Collyer H Kurkchubasche AG Bender J. Necrotizing enterocolitis and spontaneous intestinal perforation: a spatiotemporal case cluster analysis. Pediatr Qual Saf. (2019) 4:e127. 10.1097/pq9.0000000000000127 30937409 PMC6426488

[12] Neu J. Neonatal necrotizing enterocolitis: an update. Acta Paediatr Suppl. (2010) 94:100–5. 10.1111/j.1651-2227.2005.tb02163.x 16214774

[13] Eltayeb AA Mostafa MM Ibrahim NH Eltayeb AA. The role of surgery in management of necrotizing enterocolitis. Int J Surg. (2010) 8:458–61. 10.1016/j.ijsu.2010.06.005 20601251

[14] Nah SA Tan HL Tamba RP Aziz DA Azzam N. Laparoscopic localization and microlaparotomy for focal isolated perforation in necrotizing enterocolitis: an alternative approach to a challenging problem. J Pediatr Surg. (2011) 46:0–427. 10.1016/j.jpedsurg.2010.11.045 21292104

[15] Thyoka M Coppi PD Eaton S Khoo K Hall NJ Curry J. Advanced necrotizing enterocolitis part 1: mortality. Eur J Pediatr Surg. (2012) 22:008–012. 10.1055/s-0032-1306263 22434227

[16] Dukleska K Devin CL Martin AE Miller JM Sullivan KM Levy C. Necrotizing *Enterocolitis totalis*: high mortality in the absence of an aggressive surgical approach. Surgery. (2019) 165:1176–81. 10.1016/j.surg.2019.03.005 31040040

[17] Hull MA Fisher JG Gutierrez IM Jones BA Kang KH Kenny M. Mortality and management of surgical necrotizing enterocolitis in very low birth weight neonates: a prospective cohort study. J Am Coll Surg. (2014) 218:1148–55. 10.1016/j.jamcollsurg.2013.11.015 24468227

[18] Neu J Walker WA. Necrotizing enterocolitis. N Engl J Med. (2011) 364:255–64. 10.1056/NEJMra1005408 21247316 PMC3628622

[19] Gfroerer S Fiegel H Schloesser RL Rolle U. Primary Laparotomy is effective and safe in the treatment of necrotizing enterocolitis. World J Surg. (2014) 38:2730–4. 10.1007/s00268-014-2615-y 24789016

[20] Heida FH Hulscher JB Schurink M Timmer A Kooi EM Bos AF. Intestinal fatty acid-binding protein levels in necrotizing enterocolitis correlate with extent of necrotic bowel:results from a multicenter study. J Pediatr Surg. (2015) 50:1115–8. 10.1016/j.jpedsurg.2014.11.037 25783297

[21] Aydemir C Dilli D Oguz SS Ulu HO Uras N Erdeve O. Serum intestinal fatty acid binding protein level for early diagnosis and prediction of severity of necrotizing enterocolitis. Early Hum Dev. (2011) 87:659–61. 10.1016/j.earlhumdev.2011.05.004 21641735

[22] Reisinger KW Kramer BW Van der Zee DC Brouwers HA Buurman WA van Heum E. Non-invasive serum amyloid A (SAA) measurement and plasma platelets for accurate prediction of surgical intervention in severe necrotizing enterocolitis (NEC). PLoS ONE. (2014) 9:e90834. 10.1371/journal.pone.0090834 24603723 PMC3946234

[23] Cetinkaya M Ozkan H Koksal N Akaci O Ozgur T. The efficacy of serial serum amyloid a measurements for diagnosis and follow-up of necrotizing enterocolitis in premature infants. Pediatr Surg Int. (2010) 26:835–41. 10.1007/s00383-010-2635-0 20574758

